# The relationship between Iranian patients’ perception of holistic care and satisfaction with nursing care

**DOI:** 10.1186/s12912-019-0374-7

**Published:** 2019-10-26

**Authors:** Sahar Rajabpour, Masoud Rayyani, Parvin Mangolian shahrbabaki

**Affiliations:** 1 Psychiatric Nursing, Ghaem Hospital, Bardsir, Iran; 2 Community Health Nursing, Special Diseases Center, Kerman, Iran; 3 Nursing research center, Department of Critical Care Nursing, School of Nursing and Midwifery, Kerman University of Medical Sciences, Haft-bagh Highway, PO Box: 7716913555, Kerman, Iran

**Keywords:** Perception, Patient, Holistic care, Satisfaction, Nursing care

## Abstract

**Background:**

Holistic care is comprehensive care that emphasizes the interaction of human existential dimensions and has a significant role in accelerating the recovery process. Since nurses interact and communicate with patients more than other health care providers, the current study aimed to determine the Iranian patients’ perceptions of holistic care and overall satisfaction with nursing care.

**Methods:**

It is a descriptive-correlational study done on patients admitted to the oncology wards of hospitals in southeastern Iran. The holistic caring inventory and patient satisfaction instrument were used to measure the patients’ perceptions of holistic care and their satisfaction, respectively. SPSS 19 was used to analyze the data.

**Results:**

The results showed that there is a significant positive correlation between patients’ perception of holistic care and overall satisfaction with nursing care (*P* < 0.01, *r* = 032), which means that the higher the patients’ perception of holistic care, the greater their overall satisfaction. Based on the regression model, type of hospital, Patient’s perception of holistic care, education, previous experience of hospitalization, age and marriage are respectively predictors of overall satisfaction with nursing care (*P* < 0.05).

**Conclusion:**

The present study showed that patients’ overall satisfaction with nursing care depends on holistic nursing care, meaning that nurses should pay attention to patients’ physical, mental, emotional aspects and increase the quality of care.

## Background

Holistic care is comprehensive care that addresses all physical, mental, emotional, spiritual, social and economic aspects of the patient [1]. According to the definition of American Holistic Nurses Association, holistic nursing includes the care that aim to improve or promote the integrity of a person from birth to death. Florence Nightingale expanded the holistic care to nursing [2] and emphasized that the nurse should use his or her hands, heart, and power of thought in creating a therapeutic environment for the care of the body, the mind and the soul of the patient [3]. Many other nursing theoreticians such as Rogers, Newman, and Parse have taken into account this caring approach [1]. Watson believes that the separation of mind, body, and spirit is impossible, and they have an undeniable effect on each other, and holistic care helps support both the physical and non-physical aspects of the individual [4]. holistic care is the source of the nursing profession and that conscientiousness, commitment, hope, and love were integral parts of this care [5, 6].

Despite all the effects of holistic care, the studies indicate weakness in the use of holistic care and limited attention of nurses to mental, psychological and spiritual dimensions of patients [1, 7, 8]. Also, few studies have been carried out on identifying patients’ perceptions of holistic care [1, 2, 7, 9]. In most studies in developed countries where the patient’s autonomy and rights are considered, nurses believe that the cares related to emotional or psychosocial needs of patients are more important [10], but in Iran, despite a small number of studies on caring behaviors of nurses, the results of studies indicate that nurses are more interested in physical aspects of care and often neglect the psychosocial dimensions of patients, leading to patients’ dissatisfaction [11].

However, patients’ satisfaction has been recognized as one of the five indicators in quality service delivery [12, 13]. The quality of care as meeting physical needs by providing professional care, psychosocial support, satisfaction with care, and ensuring comprehensive care delivery to the patient [14, 15]. Therefore, nurses need to be trained in holistic care skills.

In Iran, due to lack of nurses, lack of time, fatigue, the managers’ interest in routine physical care, and the lack of emphasis on holistic care in nursing education, nurses may not have the necessary knowledge and skills for holistic care. For this reason, the patients are dissatisfied [12]. Peyrovi et al. (2013) reported that care for Iranian patients is focused on more physical aspects of care, while nurses are required to pay attention to other dimensions of care to provide comprehensive care [14]. It is very important and essential to study holistic nursing care for all aspects of human existential dimensions such as body, mind and soul, and the relationship between the viewpoint of patients as care recipients on holistic care and overall satisfaction with nursing care has not been studied in Iran. Therefore, the current study aimed to determine the relationship between overall patient satisfaction“ and “holistic nursing care in hospitals of southeastern Iran.

## Methods

### Study design

It is a descriptive correlational study to investigate the relationship between the Iranian patients’ perception of holistic care and overall satisfaction with nursing care in hospitals affiliated to Kerman University of Medical Sciences, which are the largest medical centers in southeastern Iran. This study lasted from July 2018 to October 2018.

### Instrument

Three questionnaires were used to collect data. First, demographic information including age, sex, marital status, level of education, employment status, economic status, length of stay at the hospital, hospitalization frequency, previous history of hospitalization and type of hospital were collected through a questionnaire that was developed by the authors and approved by 7 faculty member of nursing (Additional file 1). The participants’ perceptions of holistic care were taken by the holistic caring inventory (HCI). The holistic caring inventory was developed by Latham (1988) and instrument was evaluated by Latham (1996) in a study examining predictors of patient outcomes following caring interactions with nurses [15, 16]. This instrument includes 40 items in scaled measures the strongly disagree (score 1), disagree (score 2), agree (score 3), strongly agree (score 4). The minimum score for a holistic caring inventory is 40, and the maximum score is 160. A higher score indicates a higher perception, so score below 80 shows poor perception, between 80 and 120 is the moderate perception and above 120 is high perception. The questionnaire was measured by Gardner & Wheeler [15]. Cronbach’s alpha coefficient has been reported to be 0.88. The retest reliability coefficient was 0.89. The construct validity of this scale was 0.83 by factor analysis, which indicates the high external validity of the test. Also, the correlation ​​of items with the total test was reported to be higher than 0.50 [15, 16].

Finally, patient satisfaction instrument (PSI) was used to assess the satisfaction of participants with nursing care. Risser developed patient satisfaction instrument to evaluate the satisfaction of patients with nursing care [17], and Hinshaw, Scofield and Atwood corrected it [18]. The questionnaire includes 25 items in scaled measures including totally agree (score 5), agree (score 4), I am not sure (score 3), disagree (score 2) and totally disagree (score 1) with a minimum score of 25 and a maximum score of 125. The scores of all the items were added and then divided by 25 to calculate the mean satisfaction of the patient. A score below 58 shows dissatisfaction, between 58 and 92 is the moderate satisfaction and above 92 is complete satisfaction. The validity of this tool was determined through content validity in a study carried out at hospitals affiliated to Iran University of Medical Sciences in 2013 [14]. Also, the reliability of the tool was estimated to be 0.9 using Cronbach’s alpha coefficient in a study conducted in Iran Nursing and Midwifery Faculty in 2007 [12]. The researcher reevaluated the validity and reliability, and the Cronbach’s alpha coefficient was 0.90. Also, the content validity index was used to determine the content validity of the questionnaire and the validity of the questionnaire with a value of 0.99 was confirmed.

### Sampling and data collection

To determine the sample size, according to previous studies and correlation coefficient (0.14), 95% confidence and 90% test power, 90 samples were calculated, but to eliminate the possibility of loss of samples, 100 eligible patients admitted in the oncology wards were selected [19, 20].

After obtaining the permission of the Ethics Committee as well as permits and a letter of introduction from the Faculty of Nursing, Midwifery and Healthcare Centers, at first, two hospitals were considered similar in terms of population covered, number of nurses and patients. Then, all eligible patients who were admitted from 3 days or more in the oncology wards, were entered in the study to obtain the desired sample size. Because the shift nurses are in circulation, all patients have been cared by several nurses. The inclusion criteria included over the age of 18, the fluency in the Persian language, the length of stay from 3 days and more in the ward, no emergency medical status, and the signed consent. The sampling lasted from July 27 to October 5, and patients who did not have the consent to enter the study or were physically and mentally not able to complete the questionnaire were excluded. The data collection was done by the first researcher.

### Data analysis

In this study, descriptive and inferential statistics were used to analyze the data. The software used in this study was SPSS 19. Descriptive statistics (frequency, percentage, mean, and standard deviation) was used to describe the demographic and underlying characteristics of the research units and the mean scores of perception of the holistic care and satisfaction. The Pearson correlation coefficient was used to assess the relationship between the mean scores of patients’ perception of holistic care and overall satisfaction with nursing care because data were in normal distribution. Pearson, T-test, analysis of variance and Spearman tests were used to assess the relationship among the scores of patients’ perception of holistic care, overall satisfaction, and demographic variables. Univariate and multivariate logistic regression was used to examine the data more accurately.

## Results

According to the results of this study, of the 100 participants [50 patients from each hospital], 56% were female, 76% were married, a majority (37%) were aged between 40 and 45 years, most of them (88%) had bachelor and lower degrees and most of them [91%] were employed, 47% were economically in middle class (10,000,000 – 30,000,000 Rials), and 61% of participants were admitted for the first time. Of the 39 patients who have experienced a hospitalization, only 28% stated they had a good experience of being hospitalized, and 72% had an unpleasant and negative attitude towards hospitalization (Table 1).

**Table 1:** Demographic characteristic of the patients

Variables		Frequency	Percentage
Sex	Female	56	56
	Male	44	44
Education	PhD	2	2
	Masters	10	10
	Bachelor	44	44
	Lower than bachelor	44	44
Marital status	Married	76	76
	Single	24	24
Age	20–25	4	4
	25–30	10	10
	30–35	12	12
	35–40	12	12
	40–45	37	37
	45 years up	25	25
Employment status	Unemployed	9	9
	Employed	91	91
Monthly income	Higher than 30,000,000 Rials	12	12
	10,000,000–30,000,000 Rials	47	47
	Less than 10,000,000 Rials	41	41
Duration of admission	Less than a week	48	48
	A week	26	26
	More than a week	26	26
Admission times	First time	61	61
	The second and third times	32	32
	More than the third time	7	7
Previous experience of hospitalization	Positive	11	11
	Negative	28	28
Type of hospital	Number 1	50	50
	Number 2	50	50

The results showed that the mean score of patients’ perception of holistic care and overall satisfaction was 91 ± 3.1 and 89 ± 3.8, respectively. Therefore, according to mean scores, the patients’ perceptions of holistic care are slightly higher than average [80], and the level of overall satisfaction is moderate (between 58 and 92). According to the data, there is a significant positive correlation between the patient’s perception of holistic care and overall satisfaction with nursing care (*P* = 0.006, *r* = 0.32). Thus, with increasing patients’ perception, their level of overall satisfaction increases. The value of (0.32) suggests that the patients’ perception of holistic care explains the 10% variance of satisfaction (Table 2).

**Table 2:** Correlation between Patient’s perception of holistic care and overall patient satisfaction

Variable	N	Mean	SD	Kolmogorov-smirnov test	Pearson correlation test
Patients’ perceptions of holistic care	100	91	3.1	0.56	*r* = 0.32
Overall Patient Satisfaction	100	89	3.8	0.61	P = 0. 006

Based on the results, there is a significant positive correlation between age and the patient’s perception of holistic care (*p* < 0.01, *r* = 0.29). As age grows, perceptions of holistic care increase. There is a significant relationship between the type of hospital and perceptions of holistic care (*P* = 0. 001). Thus, perception of holistic care is higher in patients hospitalized at hospital No. 2. Also, there is a significant relationship among marriage, previous history of hospitalization, education and the patients’ perception of holistic care (*P* < 0.05). Thus, married people, those with a previously positive history of hospitalization and those with the increase in education had a higher perception of holistic care. There is no significant relationship among monthly income, length of stay at the hospital and the admission frequency of participants with their perception of holistic care.

Based on the results, there is a significant relationship between the type of hospital and overall satisfaction with nursing care (*P* = 0. 002). Thus, overall satisfaction was higher in hospital No. 2. There is a significant relationship between the history of admission, education and overall satisfaction with nursing care (*P* < 0.05). Thus, people with a positive history of hospitalization and those with the increase in education had a higher degree of overall satisfaction. According to the results, there is not a significant relationship among the economic status, length of stay at the hospital, hospitalization frequency and age with overall satisfaction.

Univariate logistic regression showed that there was a significant difference between education, employment status, marital status, previous experience, type of hospital with overall satisfaction. In order to eliminate confounding effects of independent variables and to investigate the predictors of satisfaction, all variables were entered into multivariate logistic regression model by Forward stepwise method (Likelihood Ratio). The results showed that education, employment status, marital status, previous experience and type of hospital was significantly correlated with overall satisfaction. In other words, those with PhD, Masters, and Bachelor degrees were more satisfied than those with less education. Married people were more satisfied than single people. Employees were more satisfied than non-employees. Patients with positive experience of hospitalization were more satisfied than those with negative experience, and patients with hospital number 2 were more satisfied than hospital No. 1 (Table 3).

**Table 3:** Univariate and multivariate regression model for all variables and overall satisfaction

Variable		Univariate logistic regression			Multivariate logistic regression		
		Odds ratio	Confidence Interval	*P* value	Odds ratio	Confidence Interval	*P* value
Age	20–25	1					
	25–30	1.04	1.29–0.91	0.35			
	30–35	0.82	1.45–0.22	0.28			
	35–40	1.63	1.17–0.87	0.41			
	40–45	1.24	1.41–0.65	0.74			
	45 years up	1.53	1.12–0.83	0.63			
Sex	Male	1					
	Female	1.32	0.71–2.41	0.51			
Education	PhD	1			1		
	Masters	1.22	1.59–0.91	0.01	1.57	1.07–0.97	0.001>
	Bachelor	1.09	1.15–0.84	0.02	1.31	2.85–0.29	0.001>
	Lower than bachelor	0.95	1.09–0.82	0.04	o.84	1.24–0.91	0.001>
Marital status	Married	1			1		
	Single	0.92	0.88–0.81	0.04	0.97	0.86–0.74	0.005
Employment status	Unemployed	1			1		
	Employed	2.11	2.92–0.79	0.02	3.94	2.61–1.31	0.001
Monthly income	30,000,000 Rials≥	1					
	10,000,000–30,000,000 Rials	1.38	2.09–0.47	0.32			
	≥ 10,000,000 Rials	0.79	1.85–0.24	0.43			
Duration of admission	A week	1					
	More than a week	1.76	1.09–0.51	0.69			
Admission times	First time	1					
	The second and third times	1.8	2.12–0.64	0.53			
	More than the third time	1.22	2.04–0.3	0.88			
Previous experience	Positive	1			1		
	Negative	0.51	0.79–0.1	0.03	0.58	0.24–0.01	0.004
Type of hospital	Number 1	1			1		
	Number 2	3.24	2.9–0.89	0.01	4.05	3.81–1.21	0.003

## Discussion

The current study was done on patients’ perception of holistic care, and overall satisfaction with nursing care, which showed that holistic nursing care would have an effective role in identifying the different needs of patients and their overall satisfaction with nursing care.

According to the results, the patients’ perception of holistic care is slightly higher than average. In line with these results Wolf et al.(1998) and Loh (2006) showed that patients’ perceptions of holistic care were at the moderate and higher level [21, 22]. In a different result, the study by Parvan et al. (2014) indicated that the majority of patients considered the quality of care in three psychosocial, physical and communication dimensions undesirable [23]. The possible reason for this difference with our study is that patients consider mostly the physical aspects of care, and due to lack of knowledge, they cannot accurately assess the competencies of nurses and believe that nurses are not able to predict and meet their needs. On the other hand, lack of human resources, lack of time, and reduced knowledge of nurses about holistic caring can also be other reasons for this difference.

The results showed that the mean score of patients’ overall satisfaction with nursing care was at a moderate level. The study of Gholjeh et al. (2014) is consistent with the present study [24]. It seems the patients are satisfied when they feel pleasant and relaxed with nursing cares. It is not realized unless they accept nurses emotionally and rationally. The studies by Khezri et al. (2015) and Nayeri et al. (2010) with different results showed that the majority of patients were dissatisfied with the services provided by nurses. They mentioned the reasons for patients’ dissatisfaction with nursing care such as the lack of nurses in therapeutic centers, lack of time, fatigue, high numbers of patients, the managers’ interest in routine physical care, and the lack of control over the care process and training [25, 26].

In this study, there was a significant positive correlation between the patients’ perception of holistic care and their overall satisfaction with nursing care and based on the regression model, the patients’ perception is a strong predictor for overall satisfaction. Therefore, patients’ level of perception increased with increasing overall satisfaction. In similar results, the researchers showed that patients who were more likely to observe and understand nursing care had higher levels of satisfaction with care. They showed that the implementation of a holistic care program would increase patients’ satisfaction with nursing care. They found that nurses’ behavior was fully observed by patients and patients judged the care at all times [12, 15, 21, 24]. Patients are less worried about the technical competence of nurses. In fact, recipients of health care want to receive nursing care from a nurse who listen to them and addresses their particular problems and understands them well [27–29].

Based on the results, it was observed that there is a significant relationship between the type of hospital with patients’ perception of holistic care and overall satisfaction with nursing care. The regression model also confirmed this finding, in other words, the working environment is a strong predictor of overall satisfaction. These results indicate that the type of hospital and working environment play an essential role in nursing care, perception of holistic care and satisfaction of patients. In confirming this issue, a number of studies reported that many factors such as working environment, in-service training, management quality and timeliness affects the quality of nursing care and patients’ satisfaction [30, 31].

Results showed with increasing education, patients’ overall satisfaction increase and based on the regression model, level of education is a predictor for overall satisfaction. The study of Nemati et al. (2014) reported a significant difference between the levels of satisfaction with clinical services regarding education. The satisfaction has increased with an increase in the level of education [32]. In the study of Zakeri-moghadam and Sadeghi (2013), there was a direct significant relationship between the level of education of patients and their satisfaction with nursing services. The lowest satisfaction was related to less educated patients, and the highest satisfaction was observed in patients with a diploma and higher education [33]. In a different study, Joolaee et al. (2014) showed the level of satisfaction of patients with lower education was higher than that of educated patients [34]. The possible reason for this difference with our study can be because less educated patients have a low perception of the problems and job descriptions of nurses or the satisfaction of patients might not have related to nursing care behaviors. Other factors such as structural, intra-organizational and extra-organizational factors could affect this satisfaction. The higher the expectation of people from the nurses and their care, the lower their adaptation to existing realities and, consequently, the lower their satisfaction. Because educated people have more social communications and more access to information resources, they observe existing shortages more clearly, and they are usually less satisfied.

According to the results, patients who had a previous positive experience of hospitalization were more satisfied and based on the regression model; the previous experience of hospitalization is a predictor for overall satisfaction. It seems that patients with a history of previous hospitalization recognize the health center and adjuste their expectations and are more satisfied. These findings were confirmed by a number of studies [27]. In the study of Khezri et al. (2015) there was no significant relationship between patient’s satisfaction and previous history of hospitalization [25].

Result of this study showed that there is a statistically significant relationship between age and overall satisfaction. Higher satisfaction with care was observed in older patients and age included satisfaction predictors. Several studies reported a significant difference between the levels of satisfaction with clinical services regarding age. The satisfaction has increased with an increase in age [31, 35, 36]. In different results, in the study of Williams (1998), there was no significant relationship among age and satisfaction with nursing care [19]. Also, married patients had a higher overall satisfaction and that is a predictor for satisfaction. In confirmation with these results, numerous studies have indicated that married people have more satisfaction because they have more experience and understanding of nursing care [36].

This study encountered a limitation. In this study was only a single measurement method that involved self-report questionnaire as the sole measurement method.

## Conclusion

Therefore, the hypothesis “there is a correlation between patients’ perceptions of holistic care and their satisfaction with nursing care” is acknowledged, and this reflects the increasing need of today’s societies for professional nurses and the expansion of nursing duties. Given that holistic care is a comprehensive care approach that affects all aspects of human as a whole, professional education of nursing students can play an important role in the development of competent nurses. In addition, nursing managers can provide a comprehensive view of the patient care through in-service training for staff. Since patients have the right of psychosocial and spiritual care in addition to physical one, paying attention to patients’ opinions increases the quality and quantity of health care provided by the nursing community and patient’s satisfaction. However, further studies are required on this scope.

## Supplementary information

**Additional file 1.** Is the English language versions of the demographic questionnaire developed and used in this study.

## Data Availability

Data are available by contacting the corresponding author.

## Abbreviations

HCI: Holistic Caring Inventory
PSI: Patient Satisfaction Instrument
