# Supplementary material for: The relationship between Iranian patients’ perception of holistic care and satisfaction with nursing care
Source: BMC Nurs. 2019 Oct 26;18:48. doi: 10.1186/s12912-019-0374-7 (PMC6815008; doi:10.1186/s12912-019-0374-7)
Supplement: Supplementary file 1 — Additional file 1. Is the English language versions of the demographic questionnaire developed and used in this study. [file 12912_2019_374_MOESM1_ESM.docx]

**Demographic Questionnaire**

1. **Age:** ….. yrs.
2. **Sex:**

Female 🔿 Male🔿

1. **Marital status:**

Married 🔿 Single🔿

1. **Education:**

PhD🔿 Masters🔿 Bachelor🔿 Lower than bachelor🔿

1. **Employment status:**

Unemployed🔿 Employed🔿

1. **Monthly income:**

Higher than 30000000 Rials🔿 10,000,000 – 30000000 Rials🔿 Less than 10000000 Rials🔿

1. **Duration of admission:**

Less than a week🔿 A week 🔿 More than a week🔿

1. **Admission times:**

First time🔿 The second and third times🔿 More than the third time🔿

1. **Previous experience of hospitalization:**

Positive 🔿 Negative🔿

1. **Type of hospital:**

Number 1🔿 Number 2🔿
